# The AI incident disclosure shadow: tracking AI incidents into U.S. securities filings

**DOI:** 10.3389/frai.2026.1969275

**Published:** 2026-10-21

**Authors:** Samir Chincholikar, Robin Chawla

**Affiliations:** Independent Researchers, New York, NY, United States

**Keywords:** AI Incident Database, AI risk factor disclosure, SEC filings, algorithmic accountability, securities regulation, AI governance

## Abstract

**Introduction:**

Artificial intelligence (AI) systems developed or deployed by public companies are increasingly linked to documented real-world harms, yet whether these AI incidents ever reach investors through securities filings has not been measured. Research on corporate AI transparency has focused on AI risk-factor language rather than on realized events. We provide incident-level evidence on the gap between the external record of AI incidents and U.S. securities disclosure.

**Methods:**

Using a pre-registered design, we matched 1,389 AI Incident Database (AIID) incidents dated 2019–2026 to U.S.-listed domestic filers, yielding 307 incidents involving 21 issuers. For each incident, we examined Forms 8-K, 10-K and 10-Q filed in the following 12 months and coded the strongest disclosure into four tiers: T1: incident-specific disclosure, T2: disclosure of a directly related legal consequence, T3: generic risk language on the same technology or harm family, T4: no related disclosure. Validation included a blind second-coder audit of issuer matching, a search-independent full-filing audit, and robustness checks across filing windows, issuer concentration and attribution.

**Results:**

Only 4 incidents (1.3%; 95% CI 0.4–3.3) received incident-specific disclosure, and 7 (2.3%; 0.9–4.6) received any substantive disclosure. The remaining 300 incidents (97.7%; 95.4–99.1) form an AI incident disclosure shadow. Generic AI-risk language accompanied 278 incidents, but only 84 matched both the incident’s technology and its harm. As a result, 70.4% (64.9–75.4) of incidents had at most weakly related filing language. Substantive disclosures clustered around financial-statement effects or formal legal and regulatory consequences, including reported impacts of approximately $304 million (Zillow) and $478 million (General Motors’ Cruise). In exploratory analyses, no severity gradient was detectable. The shadow rate ranged from 92.5% to 99.0% across all robustness specifications.

**Discussion:**

Securities filings routinely discuss AI risk in general terms but rarely identify realized AI incidents, so investors relying on SEC filings cannot reconstruct the external incident record. The estimates are conditional on AIID recording and issuer matching and do not imply legal materiality or noncompliance. They provide a pre-regime baseline for evaluating mandatory AI incident reporting under the EU AI Act and any future U.S. AI disclosure guidance.

## 1 Introduction

Artificial intelligence (AI) systems are increasingly embedded in products, services, operational processes, and financial decision-making. AI’s growing economic relevance and the increasing dependence of firms on algorithmic systems have been illustrated by research that documented the expanding role of AI in financial-market prediction, portfolio management, automated financial services, and auditing. At the same time, AI deployment can result in operational, legal, reputational and financial harms when systems perform unexpectedly, produce inaccurate outputs, discriminate among users or contribute to other adverse outcomes. As the economic importance of these systems grows, a critical capital markets question arises: When a publicly traded company is implicated in a reported AI incident, does that incident appear in the company’s subsequent SEC filings?

This question is different from the wider literature on the effect of AI on financial performance, forecasting, investment decisions or organizational efficiency. Previous research has largely focused on using AI to improve financial prediction, portfolio allocation, advisory services, and audit processes. Much less is known about the reverse information channel, namely how firms communicate the adverse consequences of their own development or deployment of AI to capital-market participants. The increasing availability of public incident repositories enables the monitoring of AI-related failures independent of corporate securities filings. This creates an opportunity to compare an external record of incidents with the information contained in firms’ subsequent SEC filings.

This study fills the gap at the incident level. We link incidents reported in the AI Incident Database (AIID) from 2019 to 2026 to U.S.-listed companies identified as developers or deployers of the systems in question. We then look at Forms 8-K, 10-K and 10-Q filed during the 12 months following each incident. Rather than a binary outcome, we distinguish four observable disclosure states: incident-specific disclosure (T1), disclosure of a related legal or regulatory consequence without specifically identifying the underlying AI incident (T2), generic risk language describing the same technology or harm family without identifying the event (T3), and no related disclosure found (T4). This structure enables us to separate incident-specific SEC filing disclosure from the much broader corpus of generic corporate risk language.

In the absence of an incident-specific disclosure or disclosure of a directly related legal consequence in the relevant securities filings, we describe such incidents as falling within the *AI incident disclosure shadow*. Importantly, the concept is descriptive rather than legal. The fact that an incident is included in AIID does not imply that the incident was material under securities law, that there was a disclosure obligation or that an issuer failed to comply with a legal obligation. The study therefore asks an empirical disclosure-incidence question: conditional on an externally documented AI incident involving a U.S.-listed issuer, how frequently is that event identifiable in subsequent SEC filings?

The analysis is based on three expectations. First, if securities disclosure operates through a materiality gate rather than a full incident-reporting system, incident-specific disclosure should be rare relative to the volume of publicly documented AI incidents (H1). Second, substantive disclosure should be more concentrated among incidents associated with observable financial-statement effects or formal legal and regulatory consequences (H2). This is an associational expectation, not a causal claim. Third, as an exploratory expectation, substantive disclosure need not increase monotonically with incident severity if perceived harm alone does not determine whether an event crosses a firm’s disclosure threshold (H3). H3 is treated as exploratory rather than confirmatory because the sample contains only seven substantive disclosures (Section 4.3).

This study makes several contributions. First, it provides an incident-level measure of the association between externally recorded AI incidents and subsequent securities disclosure. Second, the four-tier taxonomy distinguishes incident-specific disclosure from legal-consequence disclosure, generic coverage of same-family risks, and full non-detection, avoiding a dichotomous classification that would equate materially different disclosure patterns. Third, the analysis examines whether substantive disclosure (or lack thereof) varies by incident severity and whether the company was a developer or deployer. Separately, the financial and legal characteristics of the small number of substantively disclosed cases are analyzed. Finally, the study stresses reproducibility based on a documented entity-matching process, filing-level search records, independent reliability checks, robustness tests, a hashed pre-analysis plan and an archived replication package. Together, these features provide a baseline against which one can assess how corporate communication on AI incidents might evolve as AI governance and incident-reporting regimes develop.

The reported rates are conditional on three selection steps: an incident is recorded in AIID, an involved organization is identifiable, and that organization is resolvable to a U.S.-listed domestic filer under the study protocol. The estimates therefore describe disclosure among AIID-recorded, issuer-matchable incidents rather than corporate AI incidents generally. Section 3.1.1 formalizes this estimand and the associated selection mechanism.

## 2 Related work

*AI in the firm and capital markets*. There is a rapidly growing literature on the adoption of artificial intelligence by firms, and its implications for financial decision-making and capital markets. Recent studies in this journal include AI-based stock-price forecasting (Rohan et al., 2026), reinforcement-learning approaches to portfolio management (Yang et al., 2026), the adoption of automated and hybrid robo advisory services (Verma et al., 2026), and the use of AI to improve the quality of audit and reduce costs in banking and financial services (Johri et al., 2026). Related work associates corporate AI adoption with outcomes such as ESG performance, stock-price crash risk (Shen et al., 2025; Yang, 2026), and, in governance literature, develops frameworks for responsible AI adoption and risk management (Madanchian and Taherdoost, 2025). Cumulatively, this literature indicates that AI is becoming more economically relevant but it largely discusses adoption, performance, efficiency and governance. It provides relatively little evidence of what happens to SEC filing disclosure after a documented adverse event involving an AI system. We address this downside information channel directly.

*Regulation of materiality and disclosure*. Securities disclosure is not a complete incident reporting system. The disclosure of information is partly related to materiality, which has traditionally been defined in terms of whether there is a substantial likelihood that a reasonable investor would consider the information important, with contingent events being assessed in terms of both probability and magnitude (U.S. Supreme Court, 1976, 1988). Empirical evidence also demonstrates that disclosure mandates operate within broader institutional and enforcement environments, and can have meaningful impacts on corporate reporting behavior (Christensen et al., 2016; Leuz and Wysocki, 2016). These principles motivate an important distinction for the present study: an external observation of an AI incident does not establish that disclosure of the incident was required under securities law. We thus assess whether the reported incidents are mentioned in later filings, without making legal determinations about specific issuers or events.

*Voluntary disclosure and risk factor text*. The existing literature indicates that disclosures of corporate risk factors convey information that investors find relevant (Campbell et al., 2014; Kravet and Muslu, 2013) and that more specific disclosures are more informative than generalized disclosures (Hope et al., 2016). More generally, the filings-text literature shows how text analysis can be used to identify and classify corporate risks at scale (Loughran and McDonald, 2011; Bao and Datta, 2014). The voluntary-disclosure theory provides an alternative explanation of why firms might not disclose all adverse information. Classical models predict incentives for more disclosure (Grossman, 1981; Milgrom, 1981), but proprietary costs, reputational concerns, uncertainty and managerial incentives may interrupt this process (Dye, 1985; Verrecchia, 2001; Healy and Palepu, 2001). Also, empirical evidence indicates that managers may selectively disclose bad news or delay the disclosure of bad news (Skinner, 1994; Kothari et al., 2009; Beyer et al., 2010). We add to this literature by relating it to realized AI incidents. We differentiate between incident-specific disclosure and generic risk language that refers to the same technology or harm category, but without naming the underlying event.

*Cybersecurity disclosure*: *the closest analog, and where the analogy stops*. The closest empirical analogy is cybersecurity disclosure. Prior studies have documented publicly observable cybersecurity events preceding corporate disclosures, showing market impacts of announced breaches and disparities between actual cybersecurity events and cyber risk disclosures in securities filings (Campbell et al., 2003; Kannan et al., 2007; Gordon et al., 2006, 2010; Wang et al., 2013). Other work finds evidence that cyber-attacks can be under-reported in relation to events observable through other information channels (Amir et al., 2018). The conceptual parallel is real: both cybersecurity and AI generate operational events that can be publicly visible outside the issuer’s own reporting system, which is what makes an external incident denominator possible in either setting. We adopt that design for AI by constructing an incident-level denominator from an external incident database and then examining whether each matched event is identifiable in subsequent periodic and current reports.

The closest disclosure analog is cybersecurity, but important differences limit the comparison. Material cybersecurity incidents have a dedicated U.S. reporting architecture under Form 8-K Item 1.05 and Regulation S-K Item 106 (U.S. Securities and Exchange Commission, 2023), while state breach-notification rules provide an additional reporting layer (Romanosky et al., 2011). Cyber incidents also tend to have more clearly defined event boundaries. No comparable U.S. AI-incident reporting duty applied during the study period, although voluntary risk-management frameworks existed (Tabassi, 2023) and the EU AI Act introduces serious-incident reporting for high-risk systems (European Parliament and Council of the European Union, 2024). AI harms can also be diffuse, without the affected-person counts or remediation costs common in breach analysis, and responsibility may be distributed across a model developer, deployer and platform (Cobbe et al., 2023; OECD, 2024). These differences motivate the entity-resolution and attribution procedures in Section 3.2. In this sample, incidents that reach filings do so primarily through reported financial effects or formal legal and regulatory processes rather than through an incident-reporting duty. Cybersecurity therefore supplies the design template (an external event record compared with later filings) but not the expected disclosure rate or the attribution procedure required for AI incidents.

*AI harms, audits and incident reporting*. Another set of literatures detail taxonomies of AI harms and frameworks for auditing artificial intelligence systems (Weidinger et al., 2022; Raji et al., 2020; Bommasani et al., 2021; Costanza-Chock et al., 2022), and incident repositories such as AIID and related classification efforts have cataloged harms that have already occurred (McGregor, 2021; OECD, 2024). Further research in other safety-critical domains has shown that recorded incident rates can be significantly driven by reporting incentives, institutional design and the availability of confidential or non-punitive reporting channels (Barach and Small, 2000; Classen et al., 2011; Macrae, 2016). These literatures emphasize the significance of documenting realized harms, but they do not answer whether externally recorded AI incidents subsequently become identifiable in issuers’ SEC filings. We directly measure that connection by matching incident records to issuer filings and differentiate between incident-specific communication, disclosure of related legal consequences, generic same-family risk coverage, and cases in which no related disclosure is found.

## 3 Materials and methods

### 3.1 Study design and universe of incidents

The study uses an incident-level design that links externally documented AI incidents to subsequent securities disclosures by U.S.-listed companies. The incident universe is taken from a full snapshot of the AI Incident Database (AIID) as of 27 July 2026. The archived source file contains 1,597 incident records, of which 1,389 have incident dates between 2019 and 2026 and thus constitute the initial study population. We kept the source snapshot with a SHA-256 hash so that the underlying incident universe can be verified independently.

The unit of analysis is the single AI incident. Each AIID record was reviewed to identify the organization(s) described as developing, deploying, operating, or otherwise directly associated with the AI system in question. Then, organizations were aligned to publicly-listed issuers and where needed, resolved to corporate parents. If at least one of the implicated organizations could be linked to a U.S.-listed domestic issuer that files periodic reports with the U.S. Securities and Exchange Commission (SEC), the incident was included in the analytical sample.

The procedure resulted in 307 matched incidents for 21 issuers (Figure 1). The remaining AIID incidents were excluded because they involved private companies (*n* = 243), organizations that could not be resolved with sufficient confidence (*n* = 400), generic or individual actors rather than identifiable firms (*n* = 398), foreign-listed issuers that fell outside the study’s scope (*n* = 26), or issuers excluded under the study’s delisted-issuer rule (*n* = 15). The matched sample is concentrated at a handful of large technology firms: Alphabet accounted for 86 incidents, Meta for 81, Tesla for 36, and Microsoft for 35. The issuer-level Herfindahl–Hirschman Index was 0.18. The robustness analysis explicitly accounts for issuer concentration, which may affect estimates at the incident level.

**Figure 1:**
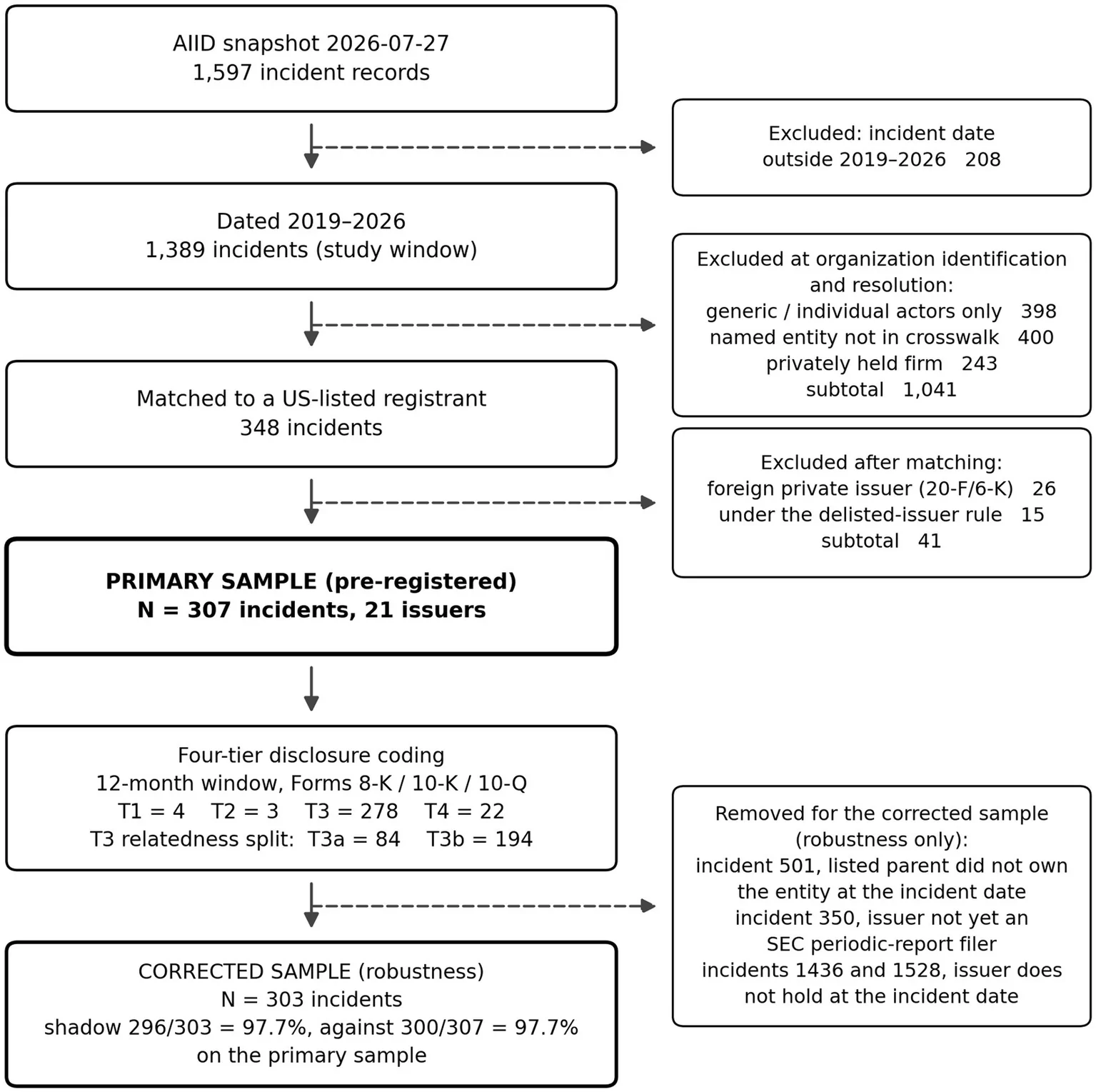
Sample construction: from 1,597 AIID incidents to the primary matchable population of *N* = 307 (21 issuers), with exclusion branches. McGregor (2021), CC BY-SA 4.0. Source: https://incidentdatabase.ai/research/snapshots/

#### 3.1.1 Estimand and selection mechanism

The quantity estimated is Pr(substantive disclosure in Forms 8-K, 10-K or 10-Q within 12 months | the incident is recorded in AIID, the organization involved is identifiable in that record, and that organization is conservatively resolvable to a U.S.-listed domestic periodic-report filer under the study protocol).

Three selection layers stand between the population of AI incidents involving listed companies and this denominator. (i) *Publicity*: AIID is media-derived, so an incident must have attracted enough public attention to be recorded. Internal failures, failures receiving little public coverage, and failures resolved without public reporting are therefore not systematically represented. (ii) *Organizational identifiability*: the AIID record must name an organization as developer or deployer, and 398 window incidents name only generic or individual actors. (iii) *Resolvability*: the named organization must map to a U.S.-listed domestic filer with sufficient confidence, which excludes private firms (243), organizations that could not be resolved confidently (400), foreign private issuers (26) and issuers excluded under the study’s delisted-issuer rule (15). Supplementary Table S4 sets out the exclusions stage by stage, with the sub-reason at each stage and the arithmetic reconciling 1,389 window incidents to the 307 analysed.

The reported rates should not be generalized to all corporate AI incidents because the denominator is selected. Incidents absent from AIID or not resolvable to a listed issuer are unobserved, and the design does not identify the direction or magnitude of the resulting selection bias. The estimand is therefore interpreted only for the AIID-recorded, issuer-matchable population described above.

### 3.2 Entity resolution and issuer matching

*Attribution criterion*. We begin with the organizations in AIID’s deployers and developers fields and map them, through a curated slug-to-registrant crosswalk, to U.S.-listed domestic filers. The developer/deployer role variable therefore inherits AIID’s attribution rather than independently establishing responsibility. It does not by itself distinguish direct operation, platform hosting or incidental mention. Section 3.2.1 adds a separate attribution-basis classification.

*One issuer per incident*. When multiple named organizations map to listed registrants, the protocol retains one issuer using the following ordering: domestic filer, domestic parent of a subsidiary, delisted issuer, then foreign private issuer; remaining ties follow AIID ordering, with deployers before developers. Twenty-seven of the 307 incidents have at least two listed-issuer candidates, 22 have at least two domestic candidates, and the maximum is five candidates for a single incident (Supplementary Table S3). Sensitivity analyses that instead favor the named developer or the listed parent change the selected issuer for 2 and 5 incidents respectively; because the alternative issuer’s filings were never searched, those incidents are dropped rather than recoded, which is why the corresponding rows of Table 1 report *N* = 305 and *N* = 302.

**Table 1:** Robustness of the shadow rate.

Specification	*n*	Shadow (95% CI)	T3b + T4	T4
Pre-registered				
Full primary sample	307	97.7% (95.4–99.1)	216	22
Issuer-clustered bootstrap	307	97.5% (93.9–99.6)	—	—
Exclude pre-2021 incidents	257	97.3%	—	—
Drop top-2 issuers	140	96.4%	—	—
Drop top-5 issuers	53	92.5%	—	—
Complete (non-right-censored) windows	252	97.2% (94.4–98.9)	—	—
6-month window	307	99.0%	—	—
12-month window (primary)	307	97.7%	216	22
18-month window	307	97.7%	—	—
24-month window	307	97.7%	—	—
Severe-tier subset	42	95.2%	21	4
Top-5-issuer subset	254	98.8%	—	—
Post-hoc, not pre-specified				
Adjudicated T2 codes	307	97.7% (95.4–99.1)	216	22
Coding-log code for incident 350	307	97.7% (95.4–99.1)	216	21
Corrected sample (crosswalk + as-of-date fixes)	303	97.7% (95.3–99.1)	212	18
Excluding platform-venue attributions	293	97.6% (95.1–99.0)	207	22
Excluding platform-venue and mentioned-only	285	97.5% (95.0–99.0)	202	19
Tie-break: prefer developer (changed incidents dropped)	305	97.7% (95.3–99.1)	215	22
Tie-break: prefer parent (changed incidents dropped)	302	97.7% (95.3–99.1)	213	20
Second-coder entity mappings	293	97.6% (95.1–99.0)	206	19

The first block reports the pre-registered analyses; the second reports post-hoc analyses that were not pre-specified. The two right-hand columns give the stricter aggregates that the T3 relatedness split makes available: T3b + T4 counts incidents whose filings carry at most weakly related language, and T4 counts those with none located. Intervals are Clopper–Pearson exact 95% unless noted. The final row drops the 14 incidents a blind second coder maps to a different issuer.

*Subsidiaries and as-of-date ownership*. Forty-seven incidents map to a listed parent through a subsidiary, product or platform slug, most commonly Waymo (17) or Cruise (12). Each parent relationship is checked as of the incident date against dated ownership records. Forty-four relationships hold, two involve in-house products with no acquisition date, and one fails: incident 501 predates UnitedHealth Group’s acquisition of naviHealth. Each ownership date is recorded with its source in the replication package.

*Crosswalk review*. The 137-row validation crosswalk was reviewed row by row, with a verdict and note recorded for each mapping. Of the 137 rows, 88 mappings stand, 40 exclusions as foreign or delisted are confirmed, 7 stand with a qualification, and 2 require correction. Together with two additional cases that fail the final as-of-date issuer check, these corrections yield a robustness sample of *N* = 303 (Table 1). Incident 103, involving Twitter while it was still listed, remains excluded under the study’s blanket delisted-issuer rule. This is conservative because it removes rather than adds an otherwise eligible incident.

*A coding-log discrepancy*. Comparing the analytical table against the per-incident coding logs finds one disagreement in 307 records: incident 350 carries T4 in the analytical table and T3 in the coding log, where a written rationale supports T3. Both tiers sit inside the shadow, so no reported rate changes, but the tier counts in Table 2 shift by one incident under the log value, and that variant is reported in Table 1.

**Table 2:** Four-tier disclosure distribution under the pre-registered coding (*N* = 307).

Tier	Meaning	*n*	Share (95% CI)
T1	Incident-specific	4	1.3% (0.4–3.3)
T2	Legal-consequence only	3	1.0% (0.2–2.8)
T3	Generic same-family risk language	278	90.6% (86.7–93.6)
T4	No related disclosure located	22	7.2% (4.5–10.6)
Substantive (T1 + T2), upper bound		7	2.3% (0.9–4.6)
Shadow (T3 + T4), summary measure		300	97.7% (95.4–99.1)

The four tiers are the primary presentation; the two aggregates below the rule are summary measures. Intervals are Clopper–Pearson exact 95%. The shadow aggregate means only that neither incident-specific nor directly related legal-consequence disclosure could be identified in Forms 8-K, 10-K and 10-Q under the study protocol.

*Reliability of issuer matching*. A blind second coder independently re-derived the issuer for a stratified sample of 80 incidents (seed 42), deliberately oversampling difficult cases: all 27 incidents with multiple listed-issuer candidates, 30 mapped through a subsidiary or product, and all 13 from issuers contributing three or fewer incidents. Agreement on issuer identity is 82.5%, with Cohen’s κ=0.79 and Gwet’s AC1 =0.82 . All 14 disagreements occur among the multi-candidate cases and none involves a T1 or T2 incident. Adjudication favors the existing mapping in ten cases and the second coder’s mapping in four. Those four reassigned incidents are incorporated into the corrected-sample robustness analysis (Supplementary Tables S8–S9; Table 1).

Therefore, the 307 incidents should be interpreted as the subset of AIID incidents that could be conservatively and reproducibly matched to U.S.-listed domestic issuers under the study protocol. The procedure does not establish that every AIID incident involving a listed company was identified, particularly where organizational identities or parent relationships were ambiguous.

#### 3.2.1 Basis for attribution

All 307 incidents are further classified by attribution basis to distinguish direct involvement from weaker association: *developer*; *deployer-operator*, where the issuer operated the system or made the deployment decision; *both*; *platform-venue*, where the issuer hosted third-party content or use but did not make the harm-producing deployment decision; and *mentioned-only*, where no development, deployment or venue relationship is evident. The classification combines AIID fields, documented override rules and a reviewed case list. The distribution is reported in Table 3, by disclosure tier in Table 4 and by issuer in Table 5. Table 1 re-estimates the main outcome after dropping platform-venue cases and, separately, both platform-venue and mentioned-only cases.

**Table 3:** Attribution basis of the 307 incidents.

Attribution basis	*n*	Share
Distribution of the 307 incidents		
Developer	49	16.0%
Deployer-operator	14	4.6%
Both	222	72.3%
Platform-venue	14	4.6%
Mentioned-only	8	2.6%

Platform-venue denotes an issuer that hosted third-party content or third-party use where the harm-producing system was not the issuer’s own deployment decision; mentioned-only denotes an issuer named without an evident development, deployment or venue relationship, including cases where the issuer is the harmed party or one of several vendors in a comparative study. No incident coded platform-venue or mentioned-only received T1 or T2 disclosure. Every case, with the rule that produced it and a confidence flag, is enumerated in AUTHOR_REVIEW.csv (Supplementary Material). Post-hoc; not pre-specified.

**Table 4:** Disclosure tier by attribution basis (adjudicated codes).

Attribution basis	*n*	T1	T2	T3a	T3b	T4
Disclosure tier by attribution basis						
Developer	49	0	0	13	33	3
Deployer-operator	14	2	0	2	7	3
Both	222	3	2	61	143	13
Platform-venue	14	0	0	5	9	0
Mentioned-only	8	0	0	3	2	3

**Table 5:** Attribution basis by issuer, ten largest contributors.

Issuer	n	Developer	Deployer-op.	Both	Venue	Mentioned
Attribution basis by issuer, ten largest contributors						
Alphabet Inc.	86	21	0	57	6	2
Meta Platforms, Inc.	81	5	2	67	7	0
Tesla, Inc.	36	6	0	30	0	0
Microsoft Corporation	35	6	1	23	0	5
Amazon.com, Inc.	16	1	3	12	0	0
General Motors Company	14	0	1	13	0	0
Apple Inc.	9	1	0	7	1	0
SoundThinking, Inc.	4	4	0	0	0	0
McDonald’s Corporation	4	0	2	2	0	0
UnitedHealth Group Inc.	3	2	0	1	0	0

### 3.3 Filing retrieval and disclosure window

SEC filings were searched beginning on the date of each matched incident and continuing for 12 months. The primary search universe is the Forms 8-K, 10-Q, and 10-K of the matched issuer for that period. The 12-month window was selected to capture both current reports and at least one additional cycle of periodic reporting, while remaining within a consistent incident-centered observation period.

Retrieval of filings was done via the issuer’s Central Index Key (CIK) and deterministic filing URLs. Searches used three families of terms to identify (1) the specific system, product, organization or incident described in the AIID, (2) the relevant artificial intelligence technology or application, and (3) the type of harm or consequence associated with the event. Search terms were recorded at the incident level. In the Risk Factors and Legal Proceedings sections, we also reviewed the first annual report following each incident, even if keyword searches did not produce a clear incident-specific match.

The search universe is deliberately restricted to Forms 8-K, 10-K and 10-Q. We do not search earnings calls, investor presentations, press releases not filed with the SEC, litigation dockets outside SEC filings, regulator announcements, corporate websites, product blogs or conference communications. An incident disclosed through one of those channels but not repeated in the specified forms is therefore coded as not identifiable in this study. The outcome is visibility in a defined SEC filing universe, not total investor-facing communication.

Search results were rated in context rather than classified only by keyword occurrence. The candidate passages were read to see if the filing was about the underlying incident, a related legal or regulatory fallout or simply a more general category of technology risk. This distinction is central to the disclosure taxonomy below.

### 3.4 Disclosure classification

Each incident was classified into one of four, mutually exclusive disclosure tiers based on the strongest disclosure that occurred within the observation window.

*T1, incident-specific disclosure*. Disclosure is tied to the AIID incident by identifying the underlying event with sufficient factual specificity. The filing does not need to use the term “artificial intelligence” if the event, system, product, or factual circumstances identify the connection.

*T2, legal-proceedings-only disclosure*. The filing reports a lawsuit, investigation, regulatory proceeding, settlement, contingency, or other formal legal consequence associated with the incident, but does not describe the underlying event as an AI incident with sufficient specificity to qualify as T1.

*T3, generic risk disclosure*. The issuer discloses the same technology or harm family implicated in the incident in the disclosure window, but does not identify the incident itself or a directly associated legal consequence.

The T3 threshold carries most of the weight in the headline result, because T3 accounts for 278 of 307 incidents, so its operational content is specified here in full. A passage qualifies as T3 only if it names, at minimum, the *class* of technology or the *class* of harm implicated in the incident. The nine technology or application families and the nine harm families used are listed in Table 6. Language that names neither class does not qualify: a statement about “technology risks,” “regulatory risks,” “litigation risks” or “operational risks” in the abstract is not T3, nor is any mention of artificial intelligence that is not tied to the implicated technology or harm class. Compensation-plan exhibits, subsidiary lists and promotional earnings-release language describing AI as an opportunity are excluded, as is the routine appearance of a product or segment name.

**Table 6:** The technology or application families and the harm families used to operationalise the T3 threshold.

Technology or application family	Harm family
Recommender and ranking systems	Misinformation and content harms
Automated content moderation	Discrimination and bias
Generative models and chatbots	Privacy and surveillance
Autonomous vehicles and driver assistance	Physical safety
Computer vision and biometrics	Economic and financial harm
Speech and natural-language processing	Intellectual property and training data
Advertising delivery and targeting	Harms to minors and self-harm
Predictive scoring, pricing and eligibility decisions	Service quality and error
Robotics and physical automation	Legal rights and due process

A filing passage qualifies as T3 only if it names at least one family from either column; the T3a/T3b split of Section 4.2 then distinguishes passages matching a family in both columns from those matching only one.

Two qualifications apply to T3. First, the protocol does not test whether generic risk language was added after the incident. T3 therefore means that the same technology or harm family was discussed during the observation window, not that the language was written because of the incident. Second, the class-level threshold remains interpretive. Section 4.2 therefore introduces a stricter split between language matching both the incident’s technology and harm families and language matching only one. Worked coding examples are provided in Supplementary Table S2.

*T4, no related disclosure found*. No incident-specific disclosure, related legal-consequence disclosure, or same-family generic risk language found in the filing universe examined per the protocol.

T1 is the primary measure of incident-specific disclosure. A broader measure of *substantive disclosure* combines T1 and T2 because both provide investors with information traceable to the event or its formal consequence. *The AI incident disclosure shadow* is T3 + T4. This definition is descriptive: it refers to incidents for which no incident-specific disclosure and no disclosure of a directly related legal consequence could be found. This does not imply that disclosure was legally required.

It is important to distinguish T3 from T4. T3 means the issuer discussed the relevant class of AI technology or harm during the observation window but did not identify the specific incident that occurred. T4 means no such language was found. T3 is therefore generic-risk coverage in the absence of incident-specific disclosure, and not evidence of intentional substitution of generic language for disclosure of the event.

*Definition*. *AI incident disclosure shadow*. An incident for which neither incident-specific disclosure (T1) nor disclosure of a directly related formal legal consequence (T2) could be identified in the issuer’s Forms 8-K, 10-K and 10-Q filed within the observation window, under this study’s search and coding protocol. The term is descriptive. It does not imply that the incident was material, that a disclosure obligation existed, that any information was withheld, or that any issuer failed to comply with a legal requirement. It means only: not identifiable in the specified SEC filing universe under the stated protocol.

### 3.5 Coding procedure and reliability

In the first pass, all 307 incidents were coded using a pre-specified codebook and decision tree. Each classification was linked to the filing evidence supporting the assigned tier. Filing evidence included filing date, form type, filing location, search terms, and relevant passage where applicable.

A second independent coding pass was performed on a stratified sample of 68 incidents. The reliability sample consisted of all T1 and T2 cases, 21 of 22 T4 cases, and a random sample of 40 T3 cases. This design intentionally oversampled the rare disclosure categories and the cases close to the substantive/non-substantive classification boundary.

Raw agreement, Cohen’s kappa (Cohen, 1960), prevalence-adjusted and bias-adjusted kappa (PABAK) and Gwet’s AC1 (Gwet, 2008) were used to evaluate agreement, and agreement is additionally reported one tier against the rest, because an aggregate coefficient can conceal a category that is unreliable on its own. We also calculated an inverse-probability-weighted agreement estimate to approximate agreement across the full incident distribution, considering that the reliability sample was stratified and not proportionally sampled. Discrepancies were noted and reconciled only after the independent coding comparison was completed.

### 3.6 Severity of incidents

The potential relationship between incident severity and substantive disclosure was also examined. For the 40 incidents for which severity information was available via the CSET AI Harm taxonomy, the existing classification was preserved (Supplementary Table S1). The other incidents were categorized as Severe, Moderate, or Limited based on a pre-specified harm-severity rubric that utilized the reported outcomes of the incident.

The final sample consisted of 42 Severe, 50 Moderate and 215 Limited incidents. Severity is an indicative analytical measure rather than an authoritative or externally validated ranking of harm, as only a subset of incidents had an external CSET classification and the remaining severity assignments were based on the study rubric. Severity was not subject to a full independent second-coding exercise as was the primary disclosure coding. This limitation is taken into account in the interpretation of the severity analysis.

### 3.7 Statistical analysis

The main estimands are the proportions of incident cases classified as T1, T1 + T2, T3, T4 and T3 + T4. Rare disclosure outcomes are reported using exact Clopper–Pearson confidence intervals (Clopper and Pearson, 1934), and Wilson intervals (Wilson, 1927) are used where appropriate for complementary descriptive estimates.

At the company level, observations are not assumed to be fully independent, because multiple incidents are associated with the same issuers. Therefore the main incident-level estimates are complemented with a 10,000-resample issuer-clustered bootstrap. The clustered estimates are interpreted with caution (limited number of issuer clusters, 21) and are reported with the exact incident level intervals, not as a replacement for the latter.

Monte Carlo exact permutation tests with 20,000 iterations were used to examine differences in substantive disclosure by severity categories and issuer roles. The asymptotic regression estimates based on the conventional approach are unstable due to sparse outcomes: there are only seven incidents in the sample that are either T1 or T2. Therefore, no confirmatory multivariable logistic model is considered the primary inferential analysis. The severity analysis tests whether the observed distribution of disclosure differs across severity groups. Failure to reject the null is interpreted as the absence of detectable evidence of a severity gradient rather than proof that severity and disclosure are exactly independent.

The four tier proportions and the T3 + T4 aggregate on the 307-incident sample are the pre-registered primary estimands. The T3a/T3b split, attribution-basis coding, as-of-date and crosswalk audits, alternative tie-breaks, severity power calculations, leave-one-positive-out role analysis and T2 adjudication are post-hoc where reported. None supersedes the pre-specified primary analysis.

All statistical analyses were performed using deterministic scripts with a fixed random seed of 42. The final analytical data set was used to independently re-derive the reported statistics, tables and figures.

### 3.8 Robustness and retrieval audits

In several analyses we investigate the sensitivity of the main estimate to a number of design choices made in the study. Alternative 6-, 18-, and 24-month filing windows were used to recalculate disclosure classifications. To address right-censoring near the end of the study window, the analysis was also repeated restricting the sample to incidents with complete follow-up periods.

Issuer concentration was assessed with leave-one-issuer-out analyses and by sequentially excluding the most incident-intensive issuers. These tests take into account whether the overall disclosure shadow is largely an artifact of a small number of companies accounting for a disproportionate share of incidents.

A separate audit of retrievals was performed to determine whether substantive disclosures were missed by the structured filing-search process. Forty incidents (20 T3 and 20 T4) were randomly selected from the non-substantive categories. All relevant filings in the observation window of each selected incident were read in full without relying on the original keyword-search procedure. The audit found no missed T1 or T2 disclosures. With no misses among the 40 audited incidents, the exact one-sided 95% upper confidence bound on the underlying miss rate is approximately 7.2%. All seven T1 and T2 classifications were also manually rechecked against the underlying filings.

### 3.9 Reproducibility and research workflow

The analytical workflow was pre-registered through a hashed pre-analysis plan prior to final estimation detailing the core sample construction, disclosure taxonomy, primary estimands, procedures for assessing reliability, and procedures for assessing robustness. The replication package includes the 307-incident matched dataset, disclosure classifications, severity coding, issuer-matching crosswalk, filing-search records, disagreement log, pre-analysis documentation, and analysis code to reproduce the reported tables, figures, and statistical results. The researchers defined the disclosure codebook and classification rules, and all entity matches, filing evidence, incident classifications, and final statistical outputs were validated. Each of the reported disclosure classifications can be traced back to the underlying source filing. The main results were independently re-derived using the final data. A generative-AI system (Anthropic Claude, Opus-class model, accessed August 2026 via the Claude Agent SDK) assisted with software scaffolding, coding and code review, and minor grammar edits, under author validation; all study design, entity resolution, incident classification, and interpretation were performed by the authors (see the Generative AI Statement).

## 4 Results

### 4.1 Primary disclosure outcomes

The final analytical sample includes 307 AIID-recorded, issuer-matchable AI incidents involving 21 U.S.-listed issuers. The four-tier distribution is the primary presentation (Table 2), and the aggregate follows it as a summary measure.

Under the pre-registered coding, 4 incidents are T1, 3 are T2, 278 are T3 and 22 are T4. Incident-specific disclosure therefore occurs in 4 of 307 incidents, 1.3% (Clopper–Pearson 95% CI: 0.4%–3.3%). Three further incidents are T2: a related legal or regulatory consequence appears in the issuer’s filings, but the underlying incident is not described with the specificity T1 requires. Generic same-family risk language without identification of the incident accounts for 278 incidents (90.6%, 86.7–93.6), and no related language was located for 22 (7.2%, 4.5–10.6).

Substantive disclosure (T1 + T2) occurs in 7 of 307 incidents, 2.3% (0.9–4.6). Because T2 has no demonstrated inter-coder reliability (Section 5.7), T1 is the primary incident-specific measure and T1 + T2 is treated as an upper bound. Re-adjudication of the three T2 cases moves the General Motors OnStar Smart Driver case from T2 to T1 and leaves two cases as T2, producing 5 T1 and 2 T2 without changing the substantive total. If the remaining two T2 cases were instead resolved to T3, the substantive count would fall to 5. Across all resolutions of T2, substantive disclosure is therefore bounded between 5 and 7 of 307 incidents (1.6–2.3%).

Aggregating the two non-substantive tiers, T3 and T4 together account for 300 of 307 incidents, an AI incident disclosure shadow of 97.7% (95.4–99.1). A bootstrap clustered by issuer gives 97.5% (93.9–99.6). Under the definition in Section 3.4 this figure means only that no incident-specific or directly related legal-consequence disclosure could be identified in Forms 8-K, 10-K and 10-Q under this protocol.

### 4.2 How related is the generic language? The T3a/T3b split

Because T3 contains 278 incidents, we further distinguish generic language that matches both the incident’s technology or application family and harm family (T3a) from generic language matching only one of the two (T3b). Ambiguous cases are assigned to T3b, making the stricter category conservative with respect to finding closely related generic coverage.

Of the 278 T3 incidents, 84 are T3a (27.4% of the full sample) and 194 are T3b (63.2%). Thus, only 30.2% of the generic-language mass matches both dimensions of the realized incident. Repeating the split with the narrower lexicon fixed before estimation yields 80 T3a cases rather than 84 (28.8% versus 30.2% of T3), indicating little sensitivity to this coding choice.

The T3a/T3b split does not affect the 97.7% shadow rate because both categories remain outside T1 and T2. It does change the interpretation of the generic-language mass: if only T3a is treated as closely related generic coverage, 216 of 307 incidents, 70.4% (64.9–75.4), have at most weakly related filing language, and 22 (7.2%) have none. The full five-way distribution is reported in Table 7.

**Table 7:** Five-way distribution under the T3 relatedness split, with adjudicated T2 codes (*N* = 307).

Tier	Meaning	*n*	Share (95% CI)
T1	Incident-specific	5	1.6% (0.5–3.8)
T2	Legal-consequence only	2	0.7% (0.1–2.3)
T3a	Generic, matches both families	84	27.4% (22.5–32.7)
T3b	Generic, matches one family only	194	63.2% (57.5–68.6)
T4	No related disclosure located	22	7.2% (4.5–10.6)
Substantive (T1 + T2), upper bound		7	2.3% (0.9–4.6)
Shadow-broad (T3a + T3b + T4) = primary		300	97.7% (95.4–99.1)
Weakly-related-or-none (T3b + T4)		216	70.4% (64.9–75.4)
None located only (T4)		22	7.2% (4.5–10.6)

T3a denotes generic filing language matching both the incident’s technology or application family and its harm family; T3b matches only one. The primary shadow rate is invariant to the T3a/T3b and T3/T4 boundaries by construction, because all three tiers sit outside the substantive categories: the split addresses the interpretation of the T3 mass, not the headline rate. Intervals are Clopper–Pearson exact 95%. Post-hoc; not pre-specified.

### 4.3 Incident severity and substantive disclosure (exploratory)

The severity analysis is exploratory because the sample contains only seven substantively disclosed incidents. Among 42 Severe incidents, 2 are T1 or T2 (4.8%, 95% CI 0.6–16.2); among 50 Moderate incidents, 1 is substantive (2.0%, 0.1–10.6); and among 215 Limited incidents, 4 are substantive (1.9%, 0.5–4.7). The corresponding T1 counts of 1, 1 and 3 are reported in Table 8.

**Table 8:** Disclosure by incident severity, exact counts (adjudicated codes).

Severity	*n*	T1	T2	T3a	T3b	T4	T1% (95% CI)	T1 + T2% (95% CI)
Severe	42	1	1	19	17	4	2.4 (0.1–12.6)	4.8 (0.6–16.2)
Moderate	50	1	0	20	26	3	2.0 (0.1–10.6)	2.0 (0.1–10.6)
Limited	215	3	1	45	151	15	1.4 (0.3–4.0)	1.9 (0.5–4.7)

Percentages with Clopper–Pearson exact 95% intervals are given for T1 and for T1 + T2 only; all intervals overlap. Monte–Carlo exact permutation test on substantive disclosure across tiers: *p* = 0.61. The design would require the Severe-group substantive rate to reach approximately 13% to detect a difference from the Limited group at 80% power, so this non-rejection carries little information. Exploratory.

A Monte Carlo exact permutation test finds no difference across severity groups ( p=0.61 ; Figure 2), but the test has little resolution. With seven substantive cases, power is 23.3% for a threefold Severe-versus-Limited rate difference and 56.1% for a fivefold difference. To achieve 80% power against the observed 1.9% Limited-group rate, the Severe-group rate would need to reach approximately 13%, about seven times the Limited-group rate. The non-rejection therefore provides little information about the existence of a smaller severity gradient.

**Figure 2:**
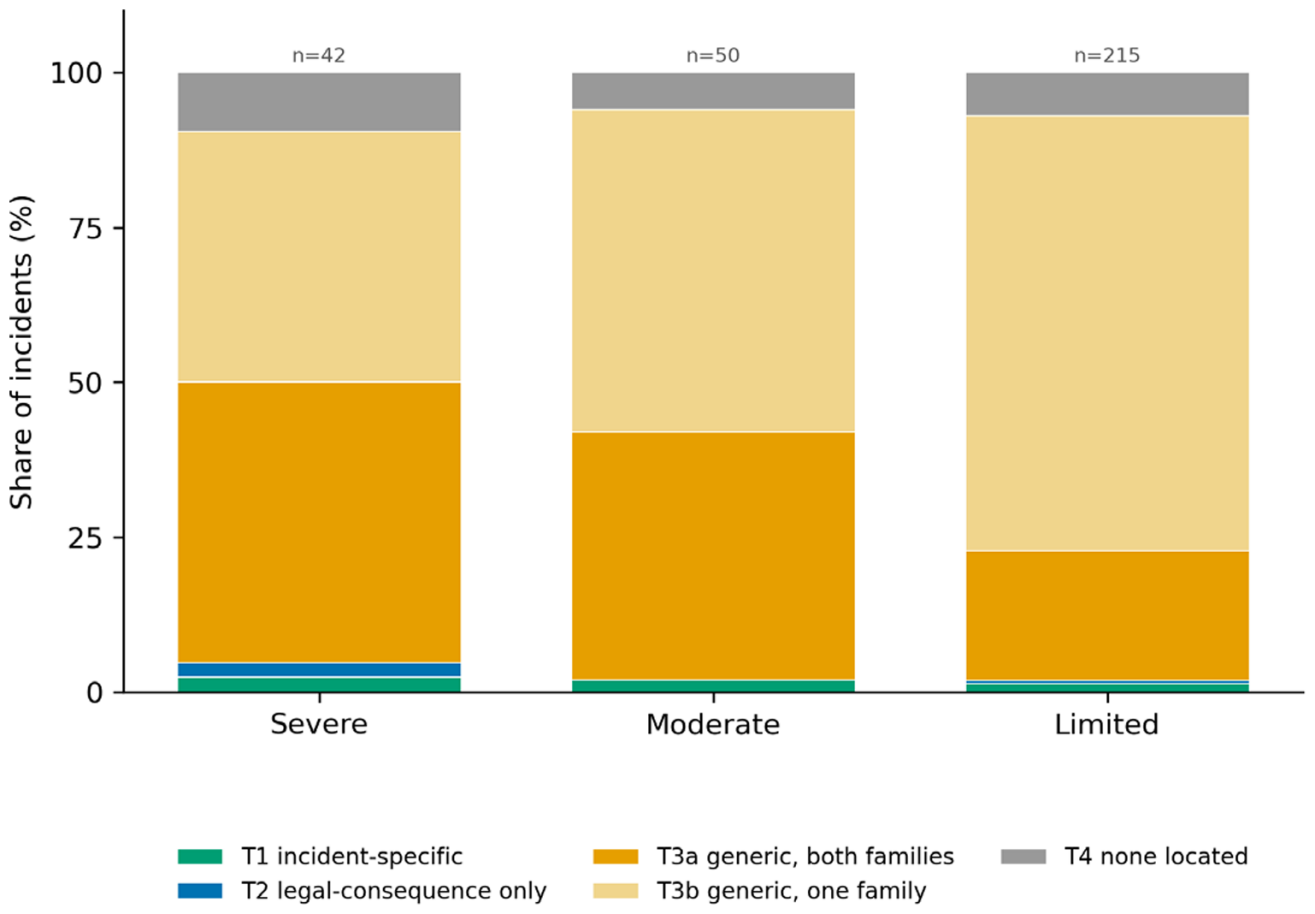
Disclosure tier by incident severity, five-way distribution with the T3 relatedness split (adjudicated codes). Bars show the share of incidents in each severity stratum falling in each tier; stratum sizes are given above each bar. The collapsed shadow rate for the same strata, with 95% Wilson intervals, is Supplementary Figure S1.

The result is therefore stated narrowly: a severity gradient is not detectable in this sample, not that severity is unrelated to disclosure. Restricting the analysis to the 40 incidents with external CSET severity classifications leaves only one substantive case, so no inferential test is conducted for that subset. Its tier distribution is reported in Supplementary Table S5.

### 4.4 Issuer role and disclosure (descriptive)

Because substantive cases are scarce, the role analysis is presented primarily descriptively. Under the adjudicated coding reported in Table 9, none of the 58 developer-only incidents receives T1 or T2 disclosure, the 14 deployer-only incidents contain 2 T1 disclosures, and the 235 dual-role incidents contain 3 T1 and 2 T2 disclosures.

**Table 9:** Issuer role against disclosure tier, exact counts (adjudicated codes).

Role	*n*	T1	T2	T3a	T3b	T4
Developer-only	58	0	0	15	38	5
Deployer-only	14	2	0	2	7	3
Both	235	3	2	67	149	14
Two-by-two contrast: substantive disclosure (T1 or T2) against all other tiers						
Developer-only	58	0 substantive, of which 0 are T1				
Any deployer (deployer-only or both)	249	7 substantive, of which 5 are T1				

The lower panel gives the two-by-two contrast the text describes. The three-way contrast rejects at conventional levels and the two-by-two does not; Supplementary Table S6 reports the leave-one-positive-out fragility of both. Descriptive; hypothesis-generating.

The three-level role contrast is statistically significant by Monte Carlo exact permutation test ( $\chi^{2}=10.434$ , p=0.024 ), whereas the two-by-two contrast between developer-only and any-deployer incidents is not ( $\chi^{2}=1.669$ , p=0.355 ). The apparent association therefore depends on retaining the small deployer-only category rather than reflecting a general developer-versus-deployer divide.

The three-level result is fragile in a leave-one-positive-out analysis (Supplementary Table S6). Across 22 perturbations of the seven substantive cases, its p -value ranges from 0.016 to 0.225 and reaches or exceeds 0.05 in 5 cases. The two-by-two contrast never rejects. Dropping or reclassifying either deployer-only positive to T3 moves the three-level p -value to approximately 0.22, while promoting one developer-only T3 case to T1 moves it to 0.054.

The defensible descriptive result is narrower: all T1 disclosures involve deployer-only or dual-role issuers, and none of the 58 developer-only incidents receives T1 or T2 disclosure. Because this pattern rests on few positive cases and a specification choice, it is treated as hypothesis-generating rather than as an estimated role effect.

### 4.5 Characteristics of events with substantive disclosures

The seven T1 or T2 incidents were concentrated in cases with observable financial-statement effects or formal legal and regulatory consequences (Table 10). Two T1 cases had large reported financial impacts. Zillow said it incurred roughly $304 million in charges to wind down its algorithm-driven business of buying and selling homes, while General Motors reported a $478 million Cruise restructuring adjustment associated with the suspension and restructuring of its autonomous-vehicle operations.

**Table 10:** All substantively disclosed incidents and their disclosure channel, ordered T1 then T2 under the pre-registered coding.

AIID id	Issuer	Tier	Filing location	Quantified financial impact
149	Zillow Group	T1	8-K Items 2.02/8.01	Yes, ≈ $304 M inventory write-down
596	General Motors	T1	10-K Items 1 and 1A	No incident-specific reserve
726	General Motors	T1	10-K Items 1 and 1A; 8-K special items	Yes—$478 M Cruise restructuring
997	Meta Platforms	T1	Legal Proceedings, named suit	No
534	Meta Platforms	T2	10-K Commitments and Contingencies	No
711	Tesla	T2	Legal Proceedings/NHTSA, DOJ matters	No
733	General Motors	T2 → T1	10-Q Commitments and Contingencies	No

Where the adjudication of Section 4.7 changed a code, both are shown as pre-registered → adjudicated; incident 733 is the one such case. Quantified financial impact records whether the filing carried a quantified financial item associated with the incident.

The remaining substantive matters were primarily litigation, regulatory proceedings, investigations or related contingencies. These included incident-specific disclosures about legal proceedings and T2 cases where the filing described the resulting legal or regulatory matter but did not identify the underlying event as an AI incident. Conversely, two of the largest incident categories in the sample, misinformation or content-related incidents (*n* = 70) and generative-AI error incidents (*n* = 57)—contained no substantive disclosures.

This concentration is consistent with H2 in a descriptive sense, with substantive disclosures concentrated among incidents associated with recognized financial-statement or formal legal reporting channels. There is no evidence that these consequences led to disclosure and the small number of positive cases does not support a formal mechanism test.

### 4.6 Issuer concentration and temporal patterns

Disclosure of substance was not evenly spread among issuers. Of the 21 issuers in the analytical sample, only 4 had one or more T1 or T2 incidents. At the same time, the relatively high overall disclosure-shadow estimate is not solely driven by the companies contributing the largest numbers of incidents (Table 1; Figure 3).

**Figure 3:**
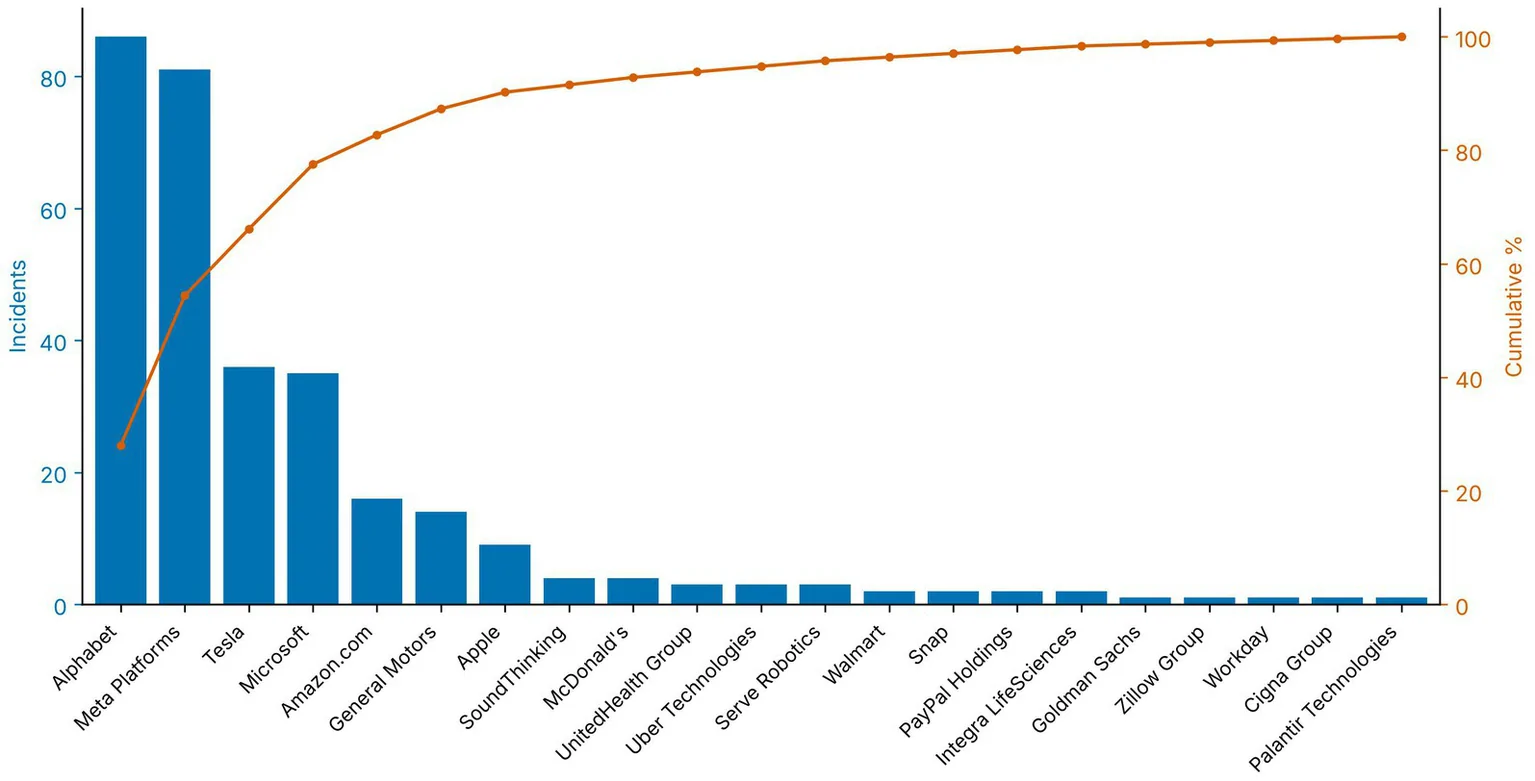
Incident concentration by issuer (Pareto): incidents per issuer and cumulative share.

In leave-one-issuer-out analysis, the estimated disclosure shadow ranged from 96.8% to 98.6%. Removing the two issuers with the most incidents, the shadow estimate fell to 96.4% and removing the five issuers with the most incidents, the estimate was 92.5%. Therefore, although issuer concentration does influence the magnitude of the estimate, the key finding is that the large majority of externally recorded incidents could not be identified via incident-specific or related legal-consequence disclosure.

We also tested whether the substantive disclosure increased over the period of the study. No systematic time increase was found. A trend test indicated *z* = −0.28 (*p* = 0.78; Figure 4), providing no evidence that the probability of substantive disclosure increased across incident years represented in the sample.

**Figure 4:**
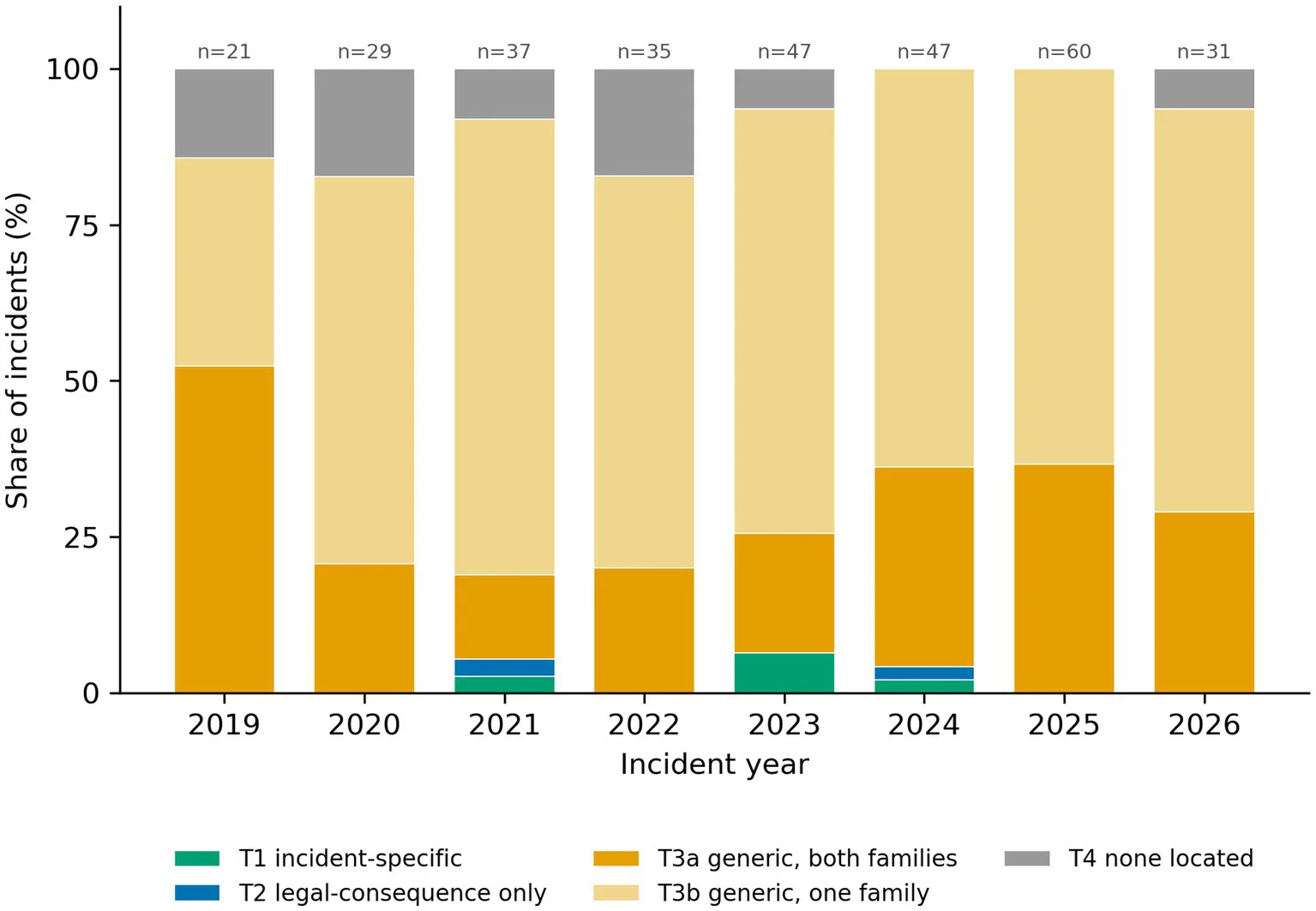
Disclosure tier by incident year (share of incidents).

### 4.7 Coding reliability and retrieval validation

The independent second-coding exercise gave raw agreement of 85.3% across the stratified reliability sample, Cohen’s kappa 0.73, prevalence-adjusted and bias-adjusted kappa 0.71, and Gwet’s AC1 0.82. The inverse-probability-weighted agreement estimate, adjusted for the deliberate oversampling of rare categories, was 90.9%. Ten disagreements arose, four of them crossing the substantive/non-substantive boundary.

Tier-specific agreement reveals one important weakness. Cohen’s kappa is 0.73 for T1, 0.76 for T3 and 0.79 for T4, but −0.023 for T2 (Supplementary Table S7): the second coder assigns the three pass-one T2 cases to T1, T3 and T4, respectively. T2 therefore has no demonstrated inter-coder reliability in this sample. For that reason, T1 is the primary incident-specific measure and T1 + T2 is reported as an upper bound. All three T2 cases were also adjudicated against the codebook, with one moving to T1 (Section 4.1).

The separate retrieval audit was a further check on the filing-search procedure. All relevant filings for 40 randomly selected non-substantive incidents (20 T3 and 20 T4 cases) were read in full and without searching. No previously missed T1 or T2 disclosures were found. The exact one-sided 95% upper confidence bound on the underlying miss rate is approximately 7.2%, with no misses among 40 audited incidents. All seven substantive disclosure cases were manually re-audited using the underlying filings.

### 4.8 Robustness to alternative specifications

The primary outcome is stable in comparison to other design choices (Table 1). Re-estimating disclosure outcomes with 6-, 18-, and 24-month filing windows did not materially alter the finding that substantive disclosure was rare and that the large majority of incidents remained in the disclosure shadow. Similarly, restricting the analysis to those incidents with complete follow-up periods also yielded similar results, suggesting that right-censoring close to the end of the study period does not explain the primary estimate.

Additional mapping and coding sensitivities are reported in Table 1. The corrected *N* = 303 sample retains a 97.7% shadow rate; excluding platform-venue incidents yields 97.6%, and excluding both platform-venue and mentioned-only incidents yields 97.5%. Alternative issuer tie-breaks leave the rate at 97.7%, while dropping every incident for which the second coder selected a different issuer yields 97.6%. T2 adjudication and the coding-log discrepancy likewise do not change the aggregate.

Across alternative disclosure windows, issuer-concentration tests, complete-follow-up restrictions, the T3a/T3b split, attribution exclusions, issuer tie-breaks, corrected mappings and the search-independent full-filing audit, the qualitative result is unchanged: incident-specific or directly related legal-consequence disclosure is rare, while generic same-family risk language is common and usually matches the realized incident along only one of the two coded dimensions.

## 5 Discussion

This study provides incident-level evidence on whether publicly documented AI incidents involving U.S.-listed companies subsequently become identifiable in securities filings. Of the 307 AI incidents by 21 issuers, only four incidents had incident-specific disclosure, and only seven had incident-specific disclosure or disclosure of legal consequence that was directly related to the incident. The resulting coverage of the AI incident disclosure shadow is 97.7% of the sample. Thus, the main finding is not that incident-specific disclosure is rare, but that realized AI incidents and SEC filing disclosure often occupy different informational layers.

Generic rather than absent risk language dominates the disclosure shadow. Of the 307 incidents, only 22 are T4 while 278 are T3. Yet the T3 split shows that this language is often only loosely related to the event as realized: 84 of 278 T3 incidents match both the technology or application family and the harm family, while 194 match only one. Under the stricter interpretation, 70.4% of all incidents have at most weakly related filing language. The central pattern is therefore not corporate silence about AI risk, but a separation between broad class-level risk discussion and identification of realized incidents. This finding does not establish managerial intent or imply that generic language was added in response to any specific event.

Several explanations for an incident’s presence in the shadow do not imply inadequate or unlawful disclosure. An event may be immaterial, may have no financial or legal consequence, may involve the issuer only indirectly, or may already be public through a channel outside this study’s filing universe. Fourteen incidents are classified as platform-venue and 8 as mentioned-only, illustrating how attribution can weaken even when an issuer appears in the incident record. More fundamentally, securities filings are not incident registers, and no general U.S. AI-incident reporting rule applied during the sample period. The shadow rate is consistent with these explanations and does not distinguish among them.

This distinction helps to clarify the relationship between risk disclosure and actual events. Incident disclosure is retrospective, pertaining to a specific event that has occurred, whereas risk-factor reporting is generally prospective and categorical. So a filing can acknowledge that a class of AI-related harm may exist without identifying every instance in that class. The results show that this difference is not small in the current sample; it accounts for the vast majority of observed incidents. Therefore, corporate AI transparency research focusing on whether firms disclose AI-related risks may substantially overstate investors’ ability to detect actual negative consequences from such disclosures.

The seven substantively disclosed cases shed more light on what situations make an AI incident identifiable in an issuer’s SEC filings. These cases are concentrated in incidents with financial statement effects or formal legal or regulatory consequences. Two incident-specific cases had associated reported financial impacts of approximately $304 million and $478 million, while several others emerged through litigation, regulatory proceedings, investigations or related contingencies. By contrast, large categories of misinformation, content-related harms, and generative-AI errors produced no substantive disclosures in the study sample.

This pattern fits H2. The evidence supports an association, although not an identified mechanism. The study cannot tell if an issuer disclosed an incident in response to accounting recognition or legal proceedings. The T2 category is also characterized by the existence of a formal legal consequence in the filing, and so the legal component of this pattern should not be viewed as an independent causal test. The more persuasive evidence is the wider spread of positive cases around incidents that made their way into established financial-reporting or legal-reporting channels, including the two T1 cases with large reported financial impacts. To test these channels independently would require a larger sample and more substantive disclosures.

The severity analysis supports only a limited conclusion: no gradient is detectable in this sample. With seven substantive disclosures, power is 23.3% even for a threefold Severe-versus-Limited rate difference, and approximately a 7.2-fold difference would be required for 80% power. The p=0.61 result should therefore not be interpreted as evidence that severity is unimportant. The mixed severity measure (40 externally classified incidents and 267 coded under the study rubric) provides an additional reason to treat this analysis as exploratory.

Issuer role shows a tentative but weak pattern. All T1 disclosures involve deployer-only or dual-role issuers, and none of the 58 developer-only incidents receives T1 or T2 disclosure. Yet the association appears in the three-level specification ( p=0.024 ) but not in the developer-only-versus-any-deployer contrast ( p=0.355 ), and single-case perturbations move the three-level result across conventional significance thresholds. The evidence therefore supports a descriptive, hypothesis-generating pattern rather than a stable role effect.

The results are consistent with a materiality-gated disclosure setting. Securities filings are not intended to be a complete repository of every operational incident related to a public company. An externally reported AI incident may be publicly salient without necessarily resulting in a financial impact, legal proceeding, or other consequence that results in identifiable SEC filing disclosure. Meanwhile, the absence of incident-specific disclosure means that investors who rely on periodic and current reports would not be able to reconstruct anything like the external incident record represented in AIID.

This has implications for the research on corporate AI transparency. Recent finance research has largely focused on AI as a productive technology for improving financial-market prediction, portfolio management, automated financial services, and auditing. In the present study, the information flow is looked at in the reverse direction. Instead of asking how AI improves financial decisions, it asks what investors learn when artificial intelligence systems themselves are the source of an adverse event. The results indicate that this downside information channel is much narrower than the broader corporate discourse on AI-related risk.

The T3 pattern is consistent with prior evidence that the informativeness of risk disclosure depends on content and specificity rather than presence alone. Wang et al. (2013) make this point for cybersecurity disclosure, while Hope et al. (2016) show more generally that specific risk-factor disclosures are more informative than generic ones. Here, broad AI-risk language is common, but it rarely identifies the realized event and usually matches it along only one coded dimension.

The robustness checks further support the main descriptive finding. Even when issuers with the most incidents are excluded, and even when alternative 6-, 18-, and 24-month disclosure windows are used, and even when the analysis is restricted to incidents with a full follow-up period, the disclosure shadow remains large. Leave-one-issuer-out estimates range from 96.8% to 98.6%, and removal of the five largest incident contributors still produces a shadow estimate of 92.5%. The results suggest that the main finding is not an artifact of a single issuer, a single observation window, or right-censoring toward the end of the sample period.

The independent coding and retrieval exercises, which also highlight areas of uncertainty, provide further support. The second coding pass revealed a high level of agreement on many reliability statistics; however, four disagreements crossed the substantive/non-substantive divide. The full-filing audit of 40 randomly selected T3 and T4 incidents identified no missed substantive disclosure. However, observing zero misses in 40 does not mean that the miss rate is zero. The one-sided 95% upper confidence bound is about 7.2%. Hence the retrieval audit supports the search protocol without precluding the possibility that some obliquely worded disclosures are still missed.

The results provide a useful baseline to compare future results against. Researchers can examine whether the relationship between externally reported incidents and SEC filing disclosure changes as AI-specific governance and incident-reporting systems are developed. Current estimates describe a period in which the frequency of AI incidents was high enough to generate a large external record, but incident-specific securities disclosure was still rare. Repeating the same incident level design under future reporting regimes allows us to separate changes in general AI risk discussion from changes in the disclosure of realized events themselves.

Most importantly, the AI incident disclosure shadow should not be seen as a sign of unlawful nondisclosure. This study does not make any determination as to whether any particular incident was legally material, whether an issuer had a duty to disclose it, or whether an issuer complied with applicable securities law. The contribution is empirical rather than adjudicative: for AI incidents independently recorded in AIID and matched to U.S.-listed issuers, a small minority became identifiable via incident-specific or directly related legal-consequence disclosure. This difference between the external incident record and the issuer’s periodic and current reports is the phenomenon documented here.

## 6 Limitations

The results should be interpreted subject to several limitations.

*Conditional denominator*. The reported rates apply to incidents that entered AIID, named an identifiable organization and could be resolved to a U.S.-listed domestic filer (Section 3.1.1). AIID is media-derived rather than a census, so internal failures and failures receiving little public coverage are not systematically represented. The 97.7% shadow rate is therefore not a population rate for corporate AI incidents, and the design does not identify the direction of selection bias.

*Filing universe*. The study measures visibility in Forms 8-K, 10-K and 10-Q, not investor-facing communication generally. Earnings calls, investor presentations, non-filed press releases, litigation dockets outside SEC filings, regulator announcements, corporate websites and conference communications are outside the search universe (Section 3.3). An incident disclosed only through one of those channels is therefore correctly classified as not identifiable for this study, but not as information unavailable to investors.

*Issuer attribution*. Linking incidents to issuers requires judgment, especially when an AIID record names multiple firms or a product or subsidiary must be linked to a parent. Twenty-seven incidents have multiple listed-issuer candidates. As-of-date ownership checks, row-level crosswalk review, attribution-basis coding and alternative tie-breaks change the aggregate by no more than 0.2 percentage points. A blind second coder agrees on 82.5% of an intentionally difficult 80-incident sample ( κ=0.79 ); all disagreements occur among multi-candidate cases and none involves T1 or T2. Residual uncertainty is therefore concentrated in attribution rather than the substantive-disclosure count.

*T2 reliability*. The legal-consequence-only category has no demonstrated inter-coder reliability in this sample: the second coder disagreed with all three pass-one T2 cases (Section 4.7). T1 is therefore treated as the primary incident-specific measure and T1 + T2 as an upper bound, with the substantive count bounded across alternative resolutions of the T2 cases.

*Issuer concentration*. The matched sample is concentrated in a relatively small number of technology companies. Alphabet, Meta, Tesla and Microsoft account for a large share of the observations and only 21 issuers are represented in total. Leave-one-issuer-out analyses, sequential exclusion of the largest incident contributors and issuer-clustered bootstrap estimates suggest these firms are not the sole drivers of the main result. However, the small number of issuer clusters limits statistical inference and the extent to which the estimated disclosure rate can be generalized to the wider population of U.S.-listed companies.

*Retrieval error*. Measurement error in filing retrieval remains possible. The language used to describe AI-related events varies greatly in media reports, corporate filings, legal proceedings and product documentation. The search protocol included incident-specific, technology-related and harm-related terms, and manual review of relevant sections of filings, but unusually indirect or oblique disclosures may have been missed. The independent full-filing audit of 40 randomly selected T3 and T4 incidents did not find any missed substantive disclosures. This provides support for the retrieval procedure. However, zero observed misses in 40 cases yields a one-sided 95% upper confidence bound of approximately 7.2%, so the audit cannot prove perfect retrieval sensitivity.

*Coding judgment*. The classification of disclosure involves an unavoidable element of judgment, especially at the boundary between disclosure of a legal consequence and general discussion of the same risk. There was substantial agreement in the independent second-coding exercise, but four disagreements crossed the primary substantive/non-substantive boundary. The full sample contains only seven substantive disclosures, so even a small number of classification differences can be proportionately important. Double coding of the entire sample would provide an even stronger reliability assessment.

*Severity*. The severity analysis is exploratory and underpowered. Power is 23.3% for a threefold Severe-versus-Limited difference, while 80% power would require approximately a 7.2-fold difference. Only 40 incidents have external CSET severity classifications; the remainder were coded using the study rubric, which was not independently double-coded. The absence of a detectable severity gradient should therefore not be interpreted as evidence of no association.

*Disclosure window*. The 12-month disclosure window is a choice of research design, not a theoretically unique interval. Some consequences of AI incidents may be fast developing. Litigation, regulatory investigations, accounting effects or organizational responses may develop over substantially longer periods. The same general conclusion holds for 6-, 18-, and 24-month windows, but there is no fixed observation window that can capture all the possible pathways of disclosure.

*T3 interpretation*. The distinction between related generic language and unrelated boilerplate remains a class-level judgment. Pre-incident presence of the same risk language was not tested, so T3 indicates only that the relevant technology or harm family was discussed during the observation window, not that the disclosure was added in response to the incident.

*Sparse outcomes*. Only seven incidents are T1 or T2, limiting multivariable inference about the determinants of substantive disclosure. The concentration of substantive cases around financial-statement effects or formal legal and regulatory consequences is therefore descriptive rather than causal, and the issuer-role association remains sensitive to specification and individual cases.

Three directions for future work follow naturally from the same incident-level design: broadening the investor-communication universe, expanding the sample enough to support multivariable analysis, and conducting cross-jurisdictional comparisons. Applying the taxonomy to earnings calls, investor presentations and press releases would distinguish non-disclosure to investors from non-repetition in periodic filings. Linking incidents to market reactions could also test whether externally reported but filing-invisible events contain investor-relevant information.

Regulatory change provides a particularly informative extension. Article 73 of the EU AI Act requires providers of high-risk AI systems to report serious incidents to national market-surveillance authorities (European Parliament and Council of the European Union, 2024), creating a mandatory incident record that can be compared with securities disclosure. Re-applying the same design under that regime, or under future U.S. AI-specific disclosure guidance, including an Item 1.05-style current-reporting approach analogous to the cybersecurity regime (U.S. Securities and Exchange Commission, 2023), would test whether mandatory incident reporting narrows the gap between externally recorded events and incidents identifiable in securities filings. The estimates reported here provide a pre-regime baseline for such comparisons.

## 7 Conclusion

This study links 307 AIID-recorded incidents involving 21 U.S.-listed issuers to subsequent Forms 8-K, 10-K and 10-Q. Under the pre-registered coding, 4 incidents receive incident-specific disclosure and 7 receive either incident-specific disclosure or disclosure of a directly related legal consequence. The resulting AI incident disclosure shadow covers 300 of 307 incidents (97.7%). These are not population rates for corporate AI incidents, but conditional estimates for incidents recorded in AIID and resolvable to a listed filer.

The shadow is not simply an absence of AI-risk language. Generic language addressing the same technology or harm family accompanies 278 incidents, but only 84 of those cases match both dimensions of the realized event. On the stricter interpretation, 70.4% of all incidents have at most weakly related filing language. Broad discussion of AI risk is therefore common even when the realized incident itself is not identifiable.

The seven substantive cases cluster around financial-statement effects or formal legal and regulatory consequences, including reported financial impacts of approximately $304 million and $478 million. By contrast, the severity and issuer-role analyses are appropriately treated as exploratory: the former is underpowered, while the latter is sensitive to specification and single-case changes.

The main descriptive result persists across alternative disclosure windows, issuer-concentration tests, attribution exclusions, issuer tie-breaks, corrected mappings and a search-independent full-filing audit. Its interpretation nevertheless remains bounded by AIID’s media-derived denominator, the limited SEC filing universe, residual ambiguity in multi-candidate issuer attribution and the lack of demonstrated reliability for T2.

The AI incident disclosure shadow is a descriptive measure, not a determination of legal materiality or compliance. It establishes a narrower fact: only a small fraction of externally recorded AI incidents that can be matched to U.S.-listed issuers are specifically identifiable in subsequent periodic and current reports. That incident-level baseline can be replicated as AI governance and incident-reporting regimes develop.

## Data Availability

The original contributions presented in the study are included in the article/Supplementary material; further inquiries can be directed to the corresponding author. Incident data were obtained from the AI Incident Database (AIID) snapshot of 27 July 2026 (https://incidentdatabase.ai/research/snapshots/), which is licensed under CC BY-SA 4.0 (McGregor, 2021). Supplementary files containing AIID-derived records are distributed under the same CC BY-SA 4.0 license. SEC filings were obtained from SEC EDGAR and are in the public domain.
