# Supplementary material for: The AI incident disclosure shadow: tracking AI incidents into U.S. securities filings
Source: Front Artif Intell. 2026 Oct 21;9:1969275. doi: 10.3389/frai.2026.1969275 (PMC13731411; doi:10.3389/frai.2026.1969275)
Supplement: Supplementary file 1 [file Data_Sheet_1.PDF]

Supplementary Material

The AI Incident Disclosure Shadow: Tracking AI Incidents into U.S. Securities Filings

Samir Chincholikar Robin Chawla

*

Supplementary Material

CSET AI-Harm severity for the externally classified subset

Supplementary Table S1 lists the 40 incidents carrying an external CSET AI-Harm classification, unchanged from the original submission. For these incidents the CSET value was preserved rather than assigned under the study rubric, and is reproduced verbatim from the released taxonomy.

Table S1: The 40 incidents carrying an external CSET AI Harm Taxonomy classification, unchanged from the original submission. The remaining 267 incidents were assigned Severe, Moderate or Limited under the pre-specified study rubric and are not listed here.

AIID incident ID	CSET classification (harm level; tangible harm)	Study severity
81	none; no tangible harm, near-miss, or issue	Moderate
82	none; no tangible harm, near-miss, or issue	Moderate
83	none; no tangible harm, near-miss, or issue	Moderate
84	none; no tangible harm, near-miss, or issue	Moderate
89	none; tangible harm definitively occurred	Severe
92	AI tangible harm event; tangible harm definitively occurred	Severe
97	AI tangible harm issue; non-imminent risk of tangible harm (an issue) occurred	Moderate
102	none; no tangible harm, near-miss, or issue	Moderate
105	AI tangible harm event; tangible harm definitively occurred	Severe
113	none; no tangible harm, near-miss, or issue	Moderate
116	AI tangible harm event; tangible harm definitively occurred	Severe
124	AI tangible harm event; tangible harm definitively occurred	Severe
125	AI tangible harm event; tangible harm definitively occurred	Severe
127	none; tangible harm definitively occurred	Severe
129	none; no tangible harm, near-miss, or issue	Moderate
139	none; no tangible harm, near-miss, or issue	Moderate
142	AI tangible harm issue; non-imminent risk of tangible harm (an issue) occurred	Moderate
143	none; no tangible harm, near-miss, or issue	Moderate
144	unclear; unclear	Limited
145	AI tangible harm issue; non-imminent risk of tangible harm (an issue) occurred	Moderate
149	AI tangible harm event; tangible harm definitively occurred	Severe
153	AI tangible harm event; tangible harm definitively occurred	Severe
156	none; tangible harm definitively occurred	Severe
157	unclear; tangible harm definitively occurred	Severe
160	AI tangible harm issue; non-imminent risk of tangible harm (an issue) occurred	Moderate
220	AI tangible harm event; tangible harm definitively occurred	Severe
281	none; non-imminent risk of tangible harm (an issue) occurred	Moderate
313	none; no tangible harm, near-miss, or issue	Moderate
354	AI tangible harm event; tangible harm definitively occurred	Severe
360	none; no tangible harm, near-miss, or issue	Moderate
367	none; no tangible harm, near-miss, or issue	Moderate
393	none; no tangible harm, near-miss, or issue	Moderate
414	none; no tangible harm, near-miss, or issue	Moderate
489	none; no tangible harm, near-miss, or issue	Moderate
501	AI tangible harm event; tangible harm definitively occurred	Severe
554	none; no tangible harm, near-miss, or issue	Moderate
574	none; no tangible harm, near-miss, or issue	Moderate
576	none; no tangible harm, near-miss, or issue	Moderate
583	none; no tangible harm, near-miss, or issue	Moderate
614	none; no tangible harm, near-miss, or issue	Moderate

T3 coding examples

The T3 threshold requires filing language that names the *class* of technology or the *class* of harm implicated in the incident. The examples below are drawn from the per-incident filing-search records in the replication package. Positive examples qualified as T3a, where the filing language matched both the incident's technology family and its harm family; negative examples qualified only as T3b, where a single family matched, which is the distinction this split isolates. Fragments are quoted verbatim from the coding record and truncated to at most forty words.

Table S2: Worked T3 coding examples, one T3a and one T3b per domain, each incident used once. *Qualified* reports which of the incident's two families the filing language matched: a T3a example matched both, a T3b example only one, which is why a T3b case does not support the reading that the generic language is closely related to the realized incident. Fragments are quoted verbatim from the filing-search record and truncated.

Domain	Split	AIID id	Issuer	Qualified (app / harm)	Verbatim fragment from the filing record
content / misinformation	T3a	1302	Alphabet	generative-llm / misinformation-content	harmful content ... violation of rights of publicity, defamation
content / misinformation	T3b	84	Meta	— / misinformation-content	negative publicity in connection with our handling of misinformation... including in connection with the COVID-19 pandemic and elections
generative-AI error	T3a	674	Alphabet	generative-llm / misinformation-content	harmful content ... may create new avenues of abuse for bad actors
generative-AI error	T3b	467	Alphabet	generative-llm / —	Our evolving AI-related efforts may give rise to risks related to harmful content, inaccuracies... and other issues.
autonomous system	T3a	181	GM	autonomous-vehicle / physical-safety	Our AV strategy is dependent upon our ability to successfully mitigate unique technological, operational and regulatory risks... To the extent accidents... associated with our autonomous driving systems occur, we could be subject to liability,
autonomous system	T3b	540	Tesla	autonomous-vehicle / —	misuse or claimed failures... of such new technologies
bias / discrimination	T3a	161	Meta	ads-targeting / discrimination-bias	changes reduce our ability to effectively target ads... adversely affected our advertising business
bias / discrimination	T3b	645	Alphabet	— / discrimination-bias	Issues in the development and use of AI may result in reputational harm.

Entity resolution detail

Table S3: Incidents in the analytical sample with more than one listed-issuer candidate. The original procedure retained one issuer per incident, selected by listing status with ties broken by the order of organizations in the AIID record; the discarded candidates were not persisted and are re-derived here.

AIID id	Date	Issuer retained	Listed candidates discarded
83	2020-10-22	Microsoft Corporation	Alphabet Inc.
92	2019-11-11	The Goldman Sachs Group Inc.	Apple Inc.
102	2020-03-23	Microsoft Corporation	Alphabet Inc.; Amazon.com, Inc.; Apple Inc.; International Business Machines
168	2022-03-01	Meta Platforms, Inc.	Alphabet Inc.; Microsoft Corporation; Netflix, Inc.; Twitter, Inc.
143	2021-02-16	Meta Platforms, Inc.	Twitter, Inc.
268	2020-03-16	Meta Platforms, Inc.	Alphabet Inc.; Twitter, Inc.
343	2021-07-11	Meta Platforms, Inc.	Twitter, Inc.
359	2021-05-23	Meta Platforms, Inc.	Twitter, Inc.
475	2021-06-02	McDonald's Corporation	International Business Machines
587	2023-05-22	Alphabet Inc.	Amazon.com, Inc.; Apple Inc.; Microsoft Corporation
718	2024-04-06	Meta Platforms, Inc.	Alphabet Inc.
731	2023-12-01	Meta Platforms, Inc.	Alphabet Inc.
734	2024-06-18	Microsoft Corporation	Alphabet Inc.; Meta Platforms, Inc.
744	2024-06-25	Microsoft Corporation	Alphabet Inc.
750	2024-07-22	Meta Platforms, Inc.	Alphabet Inc.
846	2021-10-06	Meta Platforms, Inc.	X Corp. (fmr Twitter)
859	2024-10-30	Alphabet Inc.	Meta Platforms, Inc.
881	2024-12-27	Serve Robotics Inc.	Alphabet Inc.
967	2025-03-06	Alphabet Inc.	Amazon.com, Inc.
968	2022-02-24	Microsoft Corporation	Alphabet Inc.; Meta Platforms, Inc.
1186	2025-07-31	Microsoft Corporation	Alibaba Group Holding
1188	2025-06-25	Microsoft Corporation	Alphabet Inc.
1210	2025-08-21	Alphabet Inc.	Amazon.com, Inc.
1279	2025-11-18	Microsoft Corporation	Alphabet Inc.; Meta Platforms, Inc.
1436	2022-06-23	Integra LifeSciences Holdings	Johnson & Johnson
1528	2023-05-19	Integra LifeSciences Holdings	Johnson & Johnson
1575	2026-01-20	PayPal Holdings, Inc.	Microsoft Corporation

Table S4: Stage-by-stage exclusion accounting. Of 1,389 window incidents, 1,082 are excluded and 307 remain. Note that foreign private issuers and delisted issuers are excluded *after* a successful organization-to-registrant match, which is why the mapped file contains 348 rows (307 + 26 + 15).

Stage	Exclusion	n	Sub-reason
1. Date window	outside 2019–2026	208	of 1,597 snapshot records
2. Organization named	generic or individual actors only	398	scammers, users, unnamed developers, government bodies
3. Organization resolvable	named entity not in the crosswalk	400	unresolved corporate or institutional names
4. Listed status	privately held	243	dominated by private AI labs
5. Post-match: domicile	foreign private issuer (20-F/6-K)	26	Baidu, Alibaba, Thomson Reuters and others
6. Post-match: listing	excluded under the study's delisted-issuer rule	15	Twitter/X Corp., Rite Aid
**Total excluded**		**1,082**	
**Analytical sample**		**307**	21 issuers

Severity

Table S5: Tier distribution for the 40 incidents carrying an external CSET AI-Harm severity classification. The subset contains 1 substantive disclosure, so no inferential test is performed (fewer than 5 substantive cases).

CSET severity	n	T1	T2	T3a	T3b & T4
severe	14	1	0	3	10
moderate	25	0	0	7	18
limited	1	0	0	1	0

Role analysis fragility

Table S6: Leave-one-positive-out sensitivity of the role association. Each of the seven substantive incidents is in turn dropped, reclassified one tier down, and reclassified to T3; both contrasts are recomputed by Monte-Carlo exact permutation (20,000 draws, seed 42). The final row promotes one developer-only T3 incident to T1 as an upper-bound perturbation.

Perturbation	Incident	3-way contrast		2×2 contrast	
		χ^2	p	χ^2	p
none (baseline, adjudicated)	-	10.434	0.0242	1.669	0.3552
drop	149	3.416	0.2191	1.431	0.3634
one tier down	149	10.434	0.0242	1.669	0.3552
reclassify to T3	149	3.16	0.2236	1.425	0.3650
drop	534	12.3	0.0170	1.431	0.3709
one tier down	534	12.344	0.0172	1.425	0.3649
reclassify to T3	534	12.344	0.0172	1.425	0.3649
drop	596	12.3	0.0187	1.431	0.3676
one tier down	596	10.434	0.0242	1.669	0.3552
reclassify to T3	596	12.344	0.0162	1.425	0.3628
drop	711	12.3	0.0189	1.431	0.3677
one tier down	711	12.344	0.0176	1.425	0.3631
reclassify to T3	711	12.344	0.0176	1.425	0.3631
drop	726	12.3	0.0191	1.431	0.3688
one tier down	726	10.434	0.0242	1.669	0.3552
reclassify to T3	726	12.344	0.0174	1.425	0.3680
drop	733	3.416	0.2194	1.431	0.3651
one tier down	733	10.434	0.0242	1.669	0.3552
reclassify to T3	733	3.16	0.2248	1.425	0.3650
drop	997	12.3	0.0176	1.431	0.3602
one tier down	997	10.434	0.0242	1.669	0.3552
reclassify to T3	997	12.344	0.0179	1.425	0.3665
promote one developer-only T3 incident to T1	81	7.915	0.0537	0.219	0.7079

Reliability by tier

Table S7: One-tier-against-the-rest agreement in the stratified reliability sample (n = 68), and the full confusion matrix. The T2 coefficient is negative: the second coder assigned the three pass-one T2 incidents to T1, T3 and T4 respectively, agreeing with none. T2 therefore has no demonstrated inter-coder reliability in this sample, which is why T1 is the primary measure of incident-specific disclosure and T1+T2 is reported as an upper bound.

Tier	n (pass 1)	Raw agreement	Cohen's κ
T1	4	0.971	0.734
T2	3	0.941	-0.023
T3	40	0.882	0.755
T4	21	0.912	0.793

Aggregate: raw 0.853, κ 0.726, PABAK 0.706, Gwet AC1 0.821

Pass 1 ↓ / Pass 2 →	T1	T2	T3	T4
T1	3	0	1	0
T2	1	0	1	1
T3	0	1	37	2
T4	0	0	3	18

Reliability of issuer matching

Table S8: Agreement on issuer identity between the study mapping and a blind second coder, by sampling stratum. The sample of 80 incidents (seed 42) deliberately oversamples ambiguous cases, so the overall rate is expected to be conservative relative to full-sample agreement. Overall: 82.5% raw agreement, Cohen's $\kappa = 0.79$, Gwet's AC1 = 0.82. Post-hoc; not pre-specified.

Stratum	n	Agree	Agreement
Mapped through a subsidiary or product	30	30	100.0%
More than one listed candidate	27	13	48.1%
Issuer contributing three or fewer incidents	13	13	100.0%
Single candidate, frequent issuer	10	10	100.0%
Second coder's own confidence: high	47	47	100.0%
Second coder's own confidence: low	18	11	61.1%
Second coder's own confidence: medium	15	8	53.3%

Table S9: Every disagreement on issuer identity, with its adjudication against the attribution rules of Section 3.2. Ten resolve in favour of the study mapping, four in favour of the second coder. None involves an incident coded T1 or T2. Post-hoc; not pre-specified.

Incident	Study mapping	Second coder	Adjudication
102	Microsoft	Apple	study stands
168	Meta	Netflix, Inc.	study stands
268	Meta	Alphabet	study stands
475	McDonald's Corporation	International Business Machines	study stands
587	Alphabet	Apple	study stands
718	Meta	NONE-not-listed	study stands
731	Meta	Alphabet	study stands
750	Meta	Alphabet	study stands
846	Meta	Twitter, Inc.	study stands with qualification
881	Serve Robotics	Alphabet	second coder
967	Alphabet	Amazon	study stands with qualification
1436	Integra LifeSciences Holdings	Johnson & Johnson	second coder
1528	Integra LifeSciences Holdings	Johnson & Johnson	second coder
1575	PayPal Holdings, Inc.	NONE-not-listed	second coder on basis

Shadow rate by severity

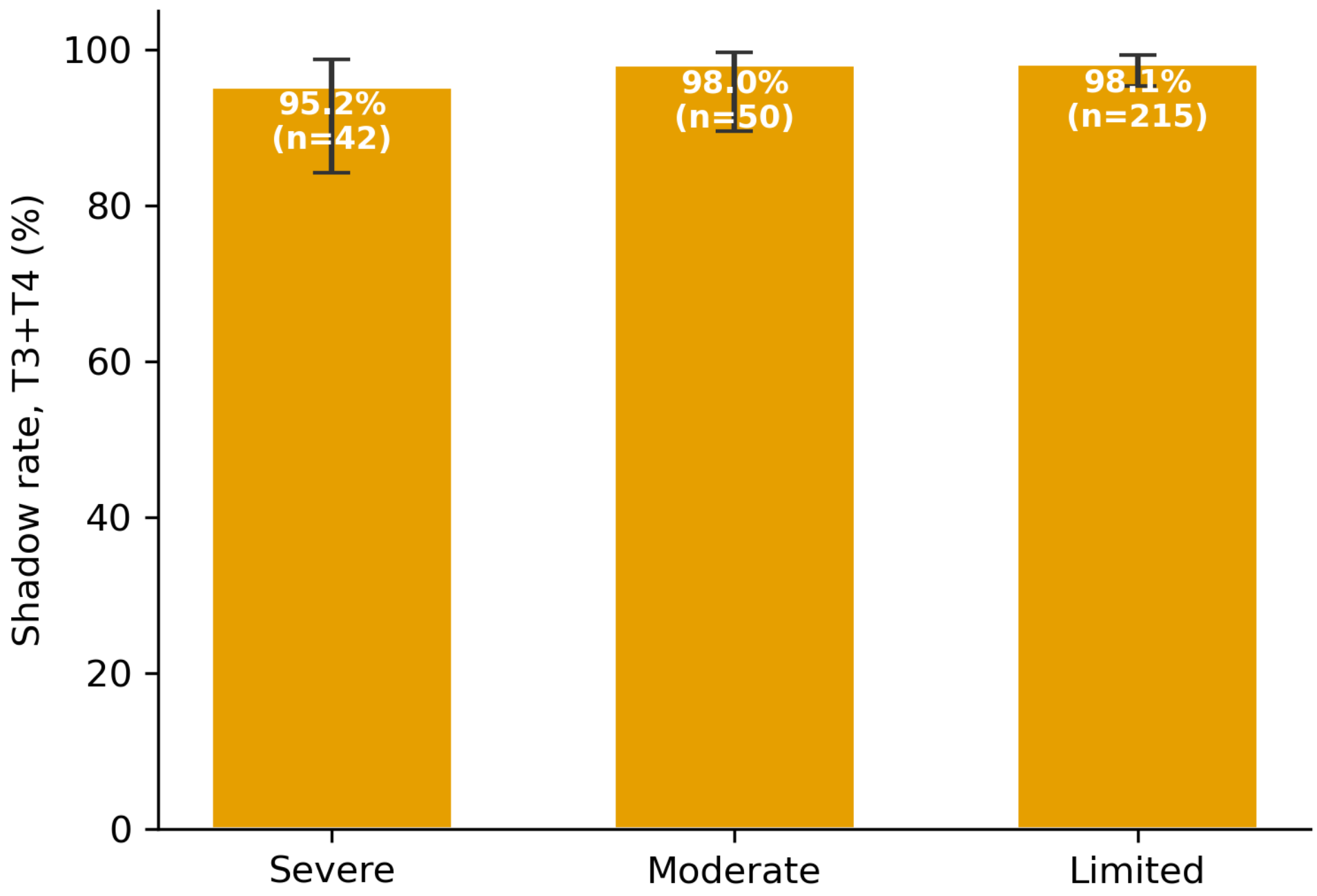

Figure S1: Collapsed shadow rate (T3+T4) by incident severity, with 95% Wilson intervals. Figure 2 of the main text gives the same strata as a five-way distribution across all tiers.
